# Surface Analyses of PVDF/NMP/[EMIM][TFSI] Solid Polymer Electrolyte

**DOI:** 10.3390/polym13162678

**Published:** 2021-08-11

**Authors:** Petr Sedlak, Dinara Sobola, Adam Gajdos, Rashid Dallaev, Alois Nebojsa, Petr Kubersky

**Affiliations:** 1 Faculty of Electrical Engineering and Communications, Brno University of Technology, Technicka 10, 616 00 Brno, Czech Republic; sobola@feec.vutbr.cz (D.S.); xgajdo12@stud.feec.vutbr.cz (A.G.); xdalla03@stud.feec.vutbr.cz (R.D.); 2 Institute of Physics of Materials, Academy of Sciences CR, Zizkova 22, 616 62 Brno, Czech Republic; 3 Central European Institute of Technology (CEITEC), Brno University of Technology, Purkynova 123, 612 00 Brno, Czech Republic; nebojsa@fme.vutbr.cz; 4 Research and Innovation Centre for Electrical Engineering (RICE), Faculty of Electrical Engineering, University of West Bohemia, Univerzitni 8, 301 00 Plzen, Czech Republic; kubersky@fel.zcu.cz

**Keywords:** solid polymer electrolyte, imidazolium ionic liquids, poly-(vinylidene fluoride), crystallinity, solvent evaporation, Raman spectroscopy, Fourier transform infrared spectroscopy, X-ray photoelectron spectroscopy, secondary ion mass spectroscopy

## Abstract

Thermal treatment conditions of solid polymer polymer electrolyte (SPE) were studied with respect to their impact on the surface morphology, phase composition and chemical composition of an imidazolium ionic-liquid-based SPE, namely PVDF/NMP/[EMIM][TFSI] electrolyte. These investigations were done using scanning electron microscopy, Raman spectroscopy, Fourier transform infrared spectroscopy, differential scanning calorimetry as well as X-ray photoelectron spectroscopy and time-of-flight secondary ion mass spectroscopy. A thoroughly mixed blend of polymer matrix, ionic liquid and solvent was deposited on a ceramic substrate and was kept at a certain temperature for a specific time in order to achieve varying crystallinity. The morphology of all the electrolytes consists of spherulites whose average diameter increases with solvent evaporation rate. Raman mapping shows that these spherulites have a semicrystalline structure and the area between them is an amorphous region. Analysis of FTIR spectra as well as Raman spectroscopy showed that the β-phase becomes dominant over other phases, while DSC technique indicated decrease of crystallinity as the solvent evaporation rate increases. XPS and ToF-SIMS indicated that the chemical composition of the surface of the SPE samples with the highest solvent evaporation rate approaches the composition of the ionic liquid.

## 1. Introduction

Solid polymer electrolytes (SPEs) could be easily and safely prepared as flexible thin films in desirable sizes with good electrochemical stability and good mechanical properties [1,2,3,4,5]. SPEs are increasingly used as electrolytes in preference to liquid electrolytes for electrochemical devices for the storage of electric energy [6,7] or sensing [8,9,10,11,12], as well as other applications. Several authors have described methods for fabricating electrochemical gas SPE-based sensors on a flexible substrate which are inexpensive and easily mass produced [8,13,14]. However, these printed gas sensors may show a certain drawbacks in a low selectivity, a low consistency of performance in short-time and long-time periods [13]. These weaknesses represent an important motivation for current research in the development of improved SPEs [14].

Generally, SPEs are solid or gel mixtures consisting of a salt dispersed in a polymer host that conducts ions through the polymer chains [15]. Alongside the most commonly studied polymer host polyethylene oxide (PEO) [4,16], polyvinylidene fluoride (PVDF) provides chemical, thermal and electrical stability, as well as unique piezoelectric and pyroelectric properties [5,17,18]. Among crystal phases types, three of them (α, β, and γ) are allowed to be steady in PVDF matrix as results of the mutual repulsion of fluorine atoms and its linear structure [19]. In polymer electrolytes, ion transport occurs predominantly in the amorphous region where the segmental motion of polymer chains becomes faster, thus the co-occurrence of crystalline and amorphous phases in PVDF-based SPEs fundamentally affects the ionic conductivity [17,19,20]. Besides techniques such as blending, addition of additives, etc. [17,18], the incorporation of an ionic liquid (IL) in the polymer matrix represents another approach that could modify overall electrochemical and mechanical properties [21]. For example, the crystallinity of polymer electrolyte is affected by the interaction of anion and cation of IL with the positive and negative polymer chains of PVDF, respectively [22,23]. It should also be noted that the electrical and mechanical properties of ionic liquids can be tailored according to their functional requirements [2,20]. These effects on crystallization and other properties of SPEs are related to the amount of IL in the polymer electrolyte [21,24,25,26]. 

Besides the IL content, thermal treatment is another way to change the properties of PVDF-based electrolytes [19,27]. Under various thermal conditions, the solvent evaporates differently from polymer mixtures and the evaporation rate has a significant influence on a degree of crystallinity that affects the morphology, chemical composition as well as ionic conductivity of the polymer electrolytes, as demonstrated on PVDF/[BMIM][TFSI] mixtures (with dimethyl formamide as solvent) [28] as well as on PVDF/[EMIM][BF4] mixtures (with NMP as solvent) [14] or even on pure PVDF with various solvents [29]. Gregorio et al. [29] showed that PVDF/NMP solutions contain predominantly the β-phase at temperatures below 100 °C, while α-phase begins to form above this temperature. Furthermore, the amount of α-phase increases as temperature is increased; however, even at 140 °C there is still a large amount of β-phase present.

This work describes research into the effects of thermal treatment conditions on the morphology of PVDF/NMP/[EMIM][TFSI] electrolyte in terms of surface morphology, phase composition, chemical composition and depth profiling using scanning electron microscopy (SEM), Raman spectroscopy, Fourier transform infrared (FT-IR), differential scanning calorimetry (DSC), X-ray photoelectron spectroscopy (XPS) and secondary ion mass spectroscopy (SIMS). Thermal treatment conditions were chosen to prepare the SPE layers in a wide range in the morphology and to get a good adhesion between the SPE layer and ceramic substrate. This paper builds on earlier research which described the effect of thermal treatment conditions on AC/DC conductivity and also current fluctuations of PVDF/IL-based solid polymer electrolyte [18] as well as on the characterization of PVDF/NMP/[EMIM][TFSI] based amperometric sensors [30,31,32,33]. 

## 2. Materials and Methods

### 2.1. Sample Preparation

The samples were prepared on an electrode platform deposited on a ceramic substrate using lift-off technology. The thickness of the golden interdigital (comb-like) electrodes varied in the order of tenths of micrometers, while the widths of the fingers and gaps were 25 µm. The solid polymer electrolyte is a mixture of three basic components: (i) 1-ethyl-3-methylimidazolium bis(trifluoromethylsulfonyl)imide ([EMIM][TFSI], Merck, Darmstadt, Germany) as ionic liquid, (ii) poly-(vinylidene fluoride) (PVDF, Lyon, Sigma-Aldrich, France) as polymer matrix, (iii) N-methyl-pyrrolidone (NMP, VWR, Fontenay-sous-Bois, France) as a solvent. The ionic liquid, polymer matrix and solvent were combined in a weight ratio 1:1:3 and mixed using a magnetic stirrer at 70 °C. Then, the blended mixture in amount of 1.5 mg was deposited by drop coating on the electrode platform, and the substrate with the SPE layer was held at a certain temperature for a specific time using a hot plate to get different microstructures and porosities in the SPE layer, i.e., to achieve different crystalline forms of the PVDF. Thermogravimetric analyses of this mixture performed previously [34] showed two well-separated processes (110 °C and 425 °C) due to the excellent thermal stability of PVDF and the ionic liquid, and the relatively low boiling point of the solvent (NMP). In this study, the four different conditions of the SPE layer preparation: (a) 80 °C for 90 s, (b) 120 °C for 90 s, (c) 120 °C for 210 s and (d) 160 °C for 600 s were predominantly chosen with regard to two factors: (i) preparation as much variation as possible in the structure and morphology of the SPE layer (associated with the particular temperature level and also with the crystallization rate of the PVDF in NMP), (ii) achievement a good adhesion between the SPE layer and the alumina substrate (associated with empirically chosen specific times for the particular temperature level). Because the thermal treatment conditions were chosen in the vicinity of the first decomposition process of the previously published thermogravimetric curve of the electrolyte [35], another issue is the residual content of the solvent (NMP) in the resultant SPE layer for different thermal treatment conditions. Standard test method for decomposition kinetics by thermogravimetry (E 1641, American Society for Testing Materials) was carried out for such estimation. Four test specimens were taken from the original mixture of electrolyte and heated through their decomposition region (25–250 °C), each of them at a different heating rate (1, 2.5, 5, and 10 °C/min). The specimen mass was recorded as a function of temperature. This standard test method allowed to estimate the mass loss of the solvent (NMP) from the electrolyte as follows: 5.5%, 32%, 58% and 100% for the following conditions (a) 80 °C 90 s, (b) 120 °C 90 s, (c) 120 °C 210 s and (d) 160 °C 600 s, respectively.

### 2.2. Scanning Electron Microscopy (SEM)

SEM images were acquired by Scanning Electron Microscope LYRA3 (TESCAN, Brno, Czech Republic) under acceleration voltage 10 kV.

### 2.3. Raman Spectroscopy

This non-destructive spectroscopic technique offers information about the molecular structure and the local chemical environments. It is also useful for studying subtle changes in electrolyte materials [35,36]. Raman spectra were collected using the confocal Raman imaging system Alpha 300 R (WITec, Ulm, Germany) with a 50 mW and 532 nm irradiation laser at 50× objective magnification. Integration time was 2 s.

### 2.4. Fourier Transform Infrared Spectroscopy

Attenuated total reflection Fourier-transform infrared spectroscopy (ATR-FTIR) is one the most used FTIR techniques since it requires minimal sample preparation for analysis and exhibits better performance. In our case, the standard FTIR was not able to distinguish the modes, while ATR-FTIR has higher measurement accuracy in the case of multicomponent samples with low reflectance signals. A Fourier infrared microscope Hyperion 3000 KIT (Bruker, Ettlingen, Germany) was used to study the phase composition. The spectra were collected in the range from 800 cm^−1^ to 4000 cm^−1^. The lower limit of the spectra is defined by the Ge crystal used.

### 2.5. Differential Scanning Calorimetry 

To describe the crystallinity of SPEs, the differential scanning calorimetry (DSC) measurements were done on DSC 204 F1 (NETZSCH, Selb, Germany) at a heating rate of 10 °C/min in temperature range from 20 °C up to 200 °C under an argon flux 20 mL/min.

### 2.6. X-ray Photoelectron Spectroscopy

Samples were investigated using an AXIS Supra X-ray photoelectron spectrometer (Kratos Analytical, Manchester, UK), with the emission current used to capture the data set at 15 mA. During the measurement process, a resolution of 80 was used to acquire wide spectra and 20 for element spectra. The angle of photoelectron take-off was 90°. The obtained XPS spectra correspond to the 6–7 nm depth analysis due to the inelastic mean free path of photoelectrons in organic compounds at kinetic energy of ∼1 keV.

### 2.7. Secondary Ion Mass Spectroscopy (SIMS)

The SIMS technique is one of the leading surface chemical analysis and imaging techniques providing molecular and elemental information in the field of material science [37]. It consists of bombarding the sample surface with an ion beam (called primary ions) and analysing the ions generated by bombardment (secondary ions). The apparatus used in our case was a TOF.SIMS 5 (Iontof, Münster, Germany) which is a system with a time of flight mass analyser that separates the ions in a field-free drift path according to their velocity. The resulting erosion speed differs depending on various factors such as material type or sputtering energy. All measurements were done in the negative mode since the samples mostly contain non-metals. The duration of the analysis for each sample was 6–10 h (from which 2–5 h are sputtering only with 2 keV Cs ions). The depth of the craters exceeds 5 μm.

## 3. Results and Discussion

**Figure 1:**
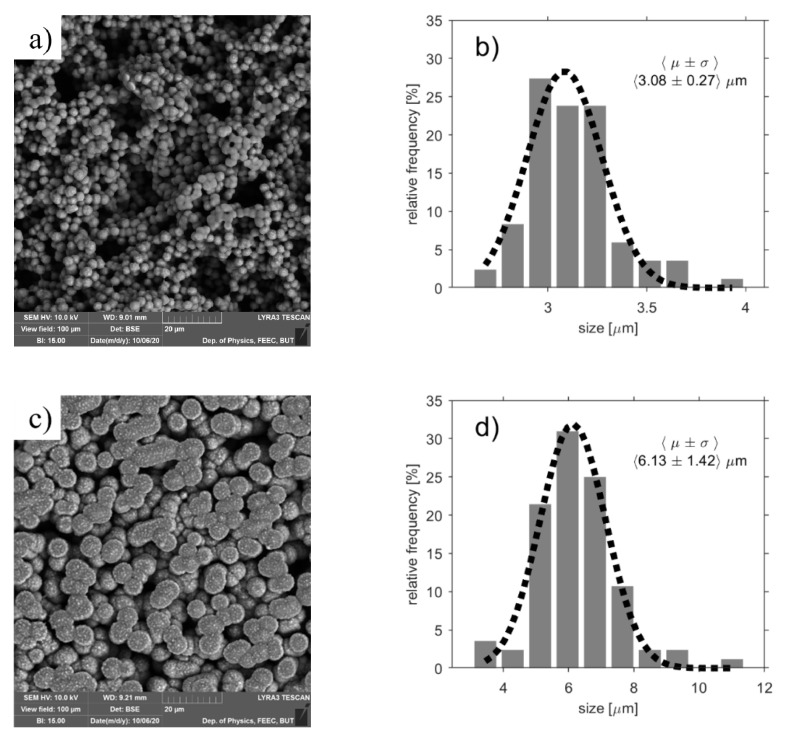

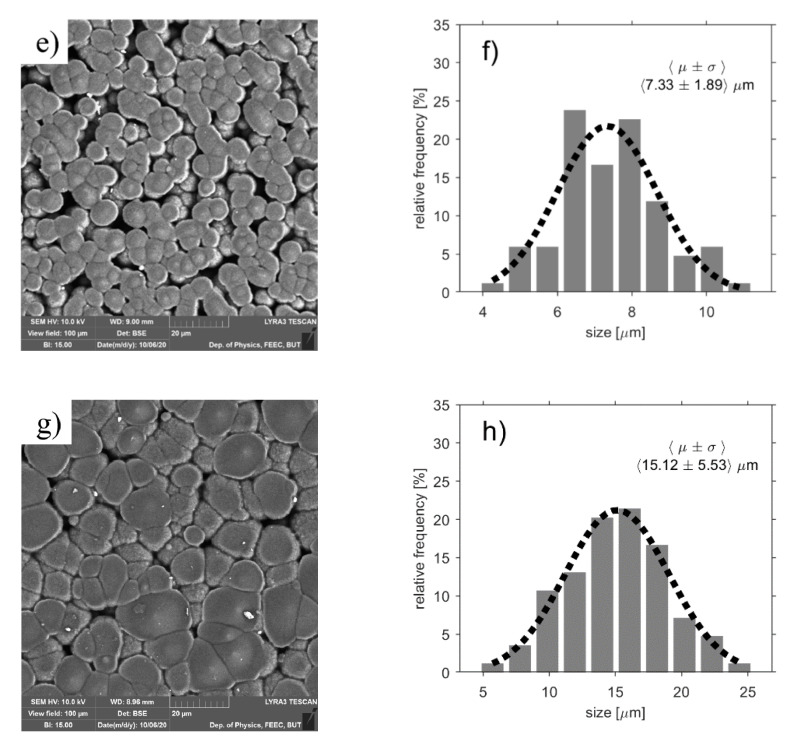
Scanning electron microscope (SEM) images and particle size distribution histograms of the studied SPEs after different preparation conditions: (**a**,**b**) 80 °C for 90 s (**c**,**d**) 120 °C for 90 s (**e**,**f**) 120 °C for 210 s (**g**,**h**) 160 °C for 600 s.

Figure 1 shows the surface of the investigated polymer electrolytes dominated by spherulites, i.e., rounded micrometric particles. Apart from the morphology of the SPEs after different preparation conditions in the middle of the SPE, Figure 1 also shows particle size distribution histograms. Each histogram is determined from analyses of four SEM images with a viewfield of 100 μm on our SEM Lyra 3 (Tescan, Brno, Czech Republic). The average diameter of SPE particles increases with preparation temperature from ~3.08 μm up to ~15.12 μm. Thus, the lower the temperature, the greater is the porosity of SPE. The porosity of the prepared layers is tightly bound to the crystallization phase, as was pointed out experimentally on mixture of the polymer (PVDF) and the solvent (NMP) [29]. The diameter of the spherulites has a non-uniform distribution across the electrolyte surface, with the spherulites in the middle of the prepared layer being larger than at the edges.

### 3.1. Raman Spectroscopy

**Figure 2:**
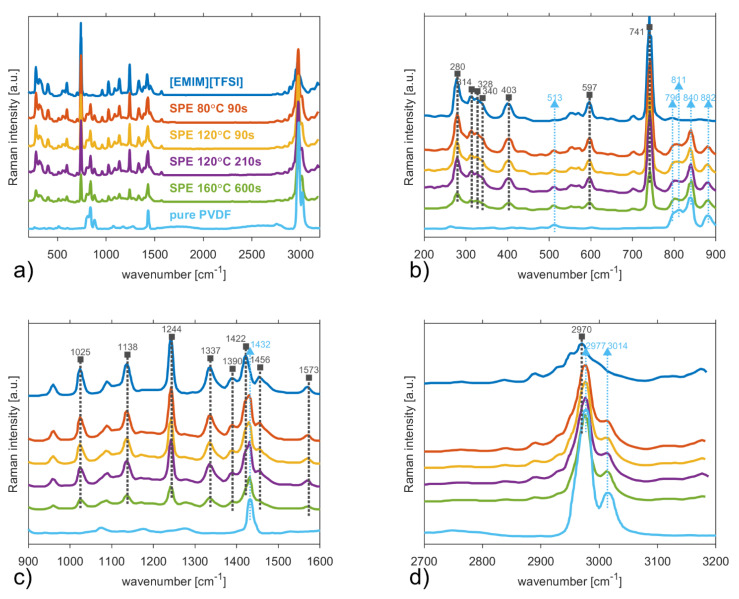
Raman spectra of ionic liquid [EMIM][TFSI], solid polymer electrolytes after four treatment conditions and PVDF in (**a**) full spectral range 200–3200 cm^−1^, (**b**) spectral range 200–900 cm^−1^, (**c**) spectral range 900–1600 cm^−1^, and (**d**) spectral range 2700–3200 cm^−1^. Grey lines and squares represent significant peaks connected to ionic liquid. Cyan lines and triangles are connected to PVDF peaks (▲◄▼).

Raman spectra from ionic liquid, PVDF and the average of each sample are presented in Figure 2. The average spectra were used for characterization because the sample consists of spherulites with different sizes and the spectra could be dependent on the acquisition point. The average data were extracted from an area of 40 × 40 um to study the Raman spectra of the samples.

Figure 2 shows the Raman spectra of the ionic liquid, PVDF as well as the four studied SPEs. Grey lines with squares and cyan lines with triangles represent significant peaks connected to the ionic liquid and PVDF respectively. The strongest Raman signals, which the solid polymers exhibit, are found in the CH stretching region (2800–3050 cm^−1^) dominated by a very strong peak line at 2977 cm^−1^ which belongs to symmetric mode of CH_2_ group [38]. The side peak at line 3014 cm^−1^ connected to β-phase asymmetric CH_2_ stretching [39] becomes more distinguishable as the temperature of SPE preparation increases. Other PVDF related lines are 513 cm^−1^ (β-phase, CF_2_ scissoring), 796 cm^−1^ (α-phase, CH_2_ rocking), 811 cm^−1^ (γ–phase, CH_2_ wagging), 840 cm^−1^ (β, CH_2_ rocking and CF_2_ stretching mode), 882 cm^−1^ (α +β + γ, CC symmetric and asymmetric stretching, CC symmetric stretching and CH_2_ twisting) and 1432 cm^−1^ (CH_2_ twisting + wagging) [38,39,40,41]. The observed band at 840 cm^−1^ is common for both β-phase and the γ-phase, however, the dominance of this peak refers only to β-phase [41,42,43,44], which was formed due to electrostatic interaction with IL.

Concerning the ionic liquid [EMIM][TFSI], a ring H–C–C–H symmetric bending, CF_3_ symmetric bending as well as contributions from two conformers of the [TFSI] anion [45,46] contribute to the second strongest Raman peak observed at line 741 cm^−1^. Further, CF_3_ symmetric bending (280 cm^−1^), S–C stretching (314, 328, and 340 cm^−1^), SO_2_ antisymmetric bending (597 cm^−1^), SO_2_ symmetric stretching (1138 cm^−1^) and a SO_2_ antisymmetric stretching with contributions from the CF_3_ symmetric stretching (1244 cm^−1^) are noticeable vibrations from [TFSI] anion. Other strong Raman peaks are assigned to CH_2_(N)/CH_3_(N) C–N stretching (1337, 1390,1422, and 1456 cm^−1^). These bands are in good agreement with published studies [45,46,47,48].

Raman mapping allows localization of PVDF phase and IL distribution. Detailed mapping was carried out using a Zeiss EC Epiplan-Neofluar DIC objective with magnification 100×. The scan area was 45 × 45 um with 90 lines and 90 points per line. Integration time was 5 sec. Excitation wavelength was 532 nm and laser power 5 mW. Formation of spherulitic texture during PVDF crystallization with the presence of IL was described earlier by polarization optical microscopy and by analysis based on the kinetics of crystallization [21]. These spherulites are composed of highly ordered lamellae which were previously confirmed by observation of a Maltese cross as fact of birefringences [49].

**Figure 3:**
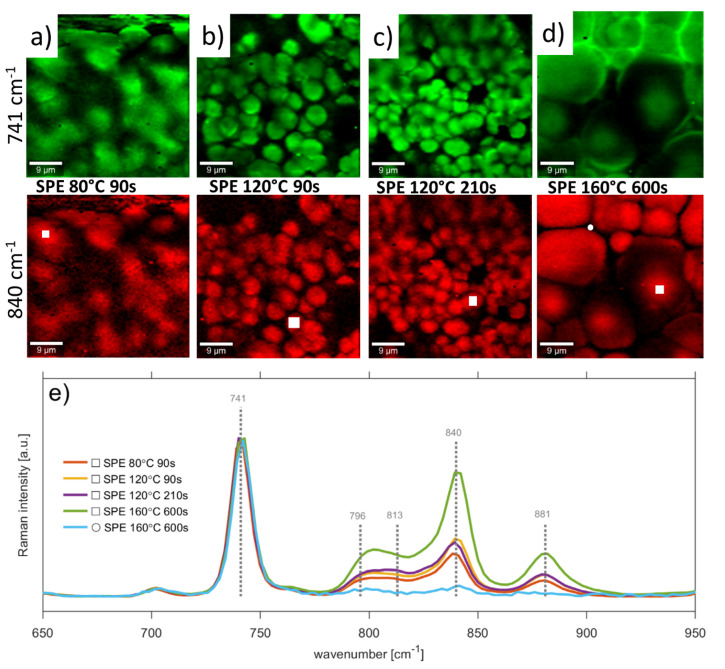
Raman maps for peaks 741 cm^−1^ (ionic liquid), 840 cm^−1^ (PVDF) for SPEs prepared at (**a**) 80 °C 90 s (**b**) 120 °C 90 s (**c**) 120 °C 210 s and (**d**) 160 °C 600 s (**e**) normalized Raman spectra corresponding to area of small white squares and white circle on SPEs. Peaks were normalized at 741 cm^−1^. White squares are located on spherulites, while circle is located between them (amorphous region).

Figure 3 shows Raman maps for peaks 741 cm^−1^ and 840 cm^−1^ as well as normalized spectra corresponding to an area of small white squares for all types of studied SPEs. According to Raman mapping of band 741 cm^−1^ in Figure 3a–d, the ionic liquid is concentrated at the edges and on the surface of the spherulites. The lamellae of the PVDF are connected by amorphous regions, which is characterized by the spectrum dominated by [EMIM][TFSI] bands as illustrated in Figure 3e for the white circle located between spherulites (Figure 3d). Thus, blue and green lines in Figure 3e are Raman spectra of the same sample (SPE 160 °C 600 s) measured at two different locations to demonstrate a semi-crystalline structure of spherulites (white square) and amorphous regions between spherulites (white circle). The normalized Raman spectra to the 741 cm^−1^ line shows in Figure 3e that the band of β-phase of PVDF (840 cm^−1^) increases with the intensity of solvent evaporation in SPE during the preparation process, and indicates that the fraction of β-phase also grows. This peak becomes more dominant over other phases, the γ-phase (811 cm^−1^) especially becomes insignificant, even to the α-phase (796 cm^−1^). The samples processed at the same temperature but different times do not show dramatic changes to the shape of Raman spectrum. The sample prepared at 120 °C for 210 s was studied under 1 V. The spectroscopy confirmed that changes occur for IL bands and not for the PVDF bands. Dependence of IL bands on electron density is confirmed by ionic type conductivity. This observation is in direct agreement with our previous study where we showed [18] that the ionic conductivity is coupled to the ionic liquid, which modifies the conductivity of the polymer matrix (PVDF).

### 3.2. Fourier Transform Infrared Spectroscopy

**Figure 4:**
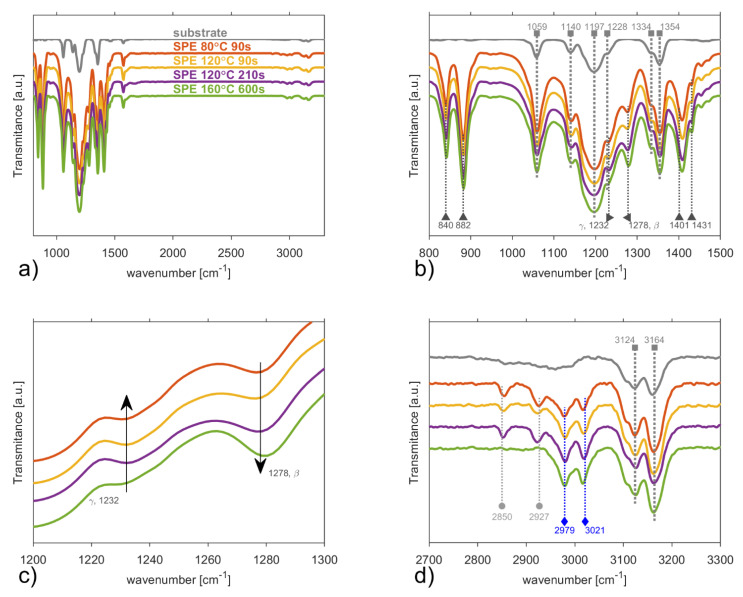
ATR-FTIR spectra of the solid polymer electrolytes after four treatment conditions and substrate in (**a**) the full spectral range 800–1430 cm^−1^, (**b**) the spectral range 800–1500 cm^−1^, (**c**) the spectral range 1200–1300 cm^−1^ of exclusive γ-phase and β-phase peaks, and (**d**) the spectral range of C–H stretching region 2700–3300 cm^−1^. Light grey squares represent peaks connected to the substrate. Grey triangles (▲◄▼) are connected to PVDF peaks. Blue diamonds represent ionic liquid peaks. Grey circles are assumed to be connected to the solvent NMP.

ATR-FTIR spectra in Figure 4a show that the C–H stretching region (2800–3300 cm^−1^) is not as dominant as in the results obtained by Raman spectroscopy. In order to separate the substrate signal, the ATR-FTIR of the ceramic substrate are also presented. Peaks at the same position for the samples and the substrate were not considered in this work. ATR-FTIR spectra in Figure 4b show the presence of exclusive peaks for γ-phase (~1232 cm^−1^) and β-phases (~1276 cm^−1^, 1431 cm^−1^) of PVDF [50,51]. The peak at 840 cm^−1^ belongs to both γ- and β-phases [52], while peaks at 880 cm^−1^ and 1401 cm^−1^ are assigned to α-, β- and γ-phases [51]. Unfortunately, the characteristic bands of the α-phase [51] (854, 975, 1149, 1209, 1383 and 1423 cm^−1^) are weak or located below (410, 489, 532, 614, 763, 795 cm^−1^) the lower limit of our apparatus, which is given by the Ge crystal used. Thus, the fractions of a particular PVDF phase cannot be estimated by the generally accepted approach [51,53,54,55] based on the α-phase peak at 763 cm^−1^. In order to calculate the relative percentage of γ- and β-phases we consider the number of electroactive phases consist only of γ- and β-phases (100%) and use the peak to valley method [51]: valley of ~1276 cm^−1^ and peak of 1260 cm^−1^ for β-phase and valley of ~1232 cm^−1^ and peak of 1225 cm^−1^ for γ-phase. Table 1 shows relative percentage of γ- and β-phases for all samples.

Electrostatic interaction between the ionic liquid and the PVDF polymeric chains leads to crystallization of PVDF into the electroactive β-phase. Increasing the temperature leads to a shift of vibration modes from ~1276 cm^−1^ (for SPE 80 °C 90 s) to ~1279 cm^−1^ (SPE 160 °C 600 s) and from ~1230 cm^−1^ (for SPE 80 °C 90 s) to ~1232 cm^−1^ (for SPE 160 °C 600 s). This shift to higher wavenumbers can be attributed to the size effect of the crystallites [56] and the firm interaction of the polymer matrix with the IL cation (more precisely, between CF_2_ groups of PVDF with the imidazolium ring) [57,58]. The higher intensity of SPE preparation conditions (temperature and time) leads to the higher evaporation rate of NMP [18,34] as well as to an increase of the PVDF β-phase in the SPE, as indicated in Table 1 which is in full agreement with other experimental studies [28]. 

FTIR spectra of the C–H stretching region (2800–3300 cm^−1^) are shown in Figure 4d. Ionic liquid is represented by two characteristic peaks of (N)CH_3_ at 2979 cm^−1^ [59] and C(2)–H 3018 cm^−1^ [60], which are present for all samples. The peaks of C–H vibration at ~2921 cm^−1^ (-CH_3_) at 2853 cm^−1^ (-CH_2_-) could be assigned to aliphatic carbon–hydrogen stretching in methyl and methylene groups of NMP [61] and are not observed for the sample (SPE 160 °C 600 s) [62,63,64]. The disappearance of these two peaks could be explained by the removal of NMP in the last sample.

### 3.3. Differential Scanning Calorimetry

**Figure 5:**
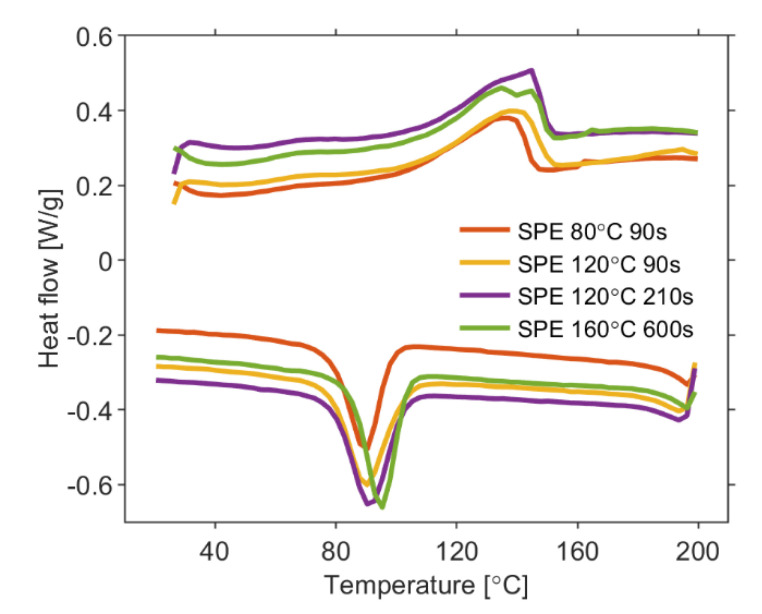
DSC thermogram of PVDF/NMP/[EMIM][TFSI] electrolytes of four treatment conditions.

DSC analysis were carried out on all types of the solid polymer electrolytes to investigate their relative crystallinity. Figure 5 shows the DSC curves where the melting temperature around 140 °C and the crystallization temperature around 90 °C are observed for each electrolyte under investigation. These temperatures increase as solvent evaporation rate increases for particular preparation conditions of SPE. The temperature difference between melting and crystallization is connected to the kinetics of crystallization, i.e., polymer chains reorganization and recrystallization [27]. 

Assuming pure PVDF to be 100%, the relative crystallinity, γ_c_, is determined by formula [65]

(1) $$\gamma_{c}=\frac{\Delta H_{f}}{\Delta H_{f0}\times \varphi }\times 100\;\left[\%\right],$$

where ∆*H*_f_, the enthalpy of fusion, was determined as area below melting peaks, ∆*H*_f0_ represents the heat of fusion for the pure crystalline PVDF (for this case 104.7 J/g [27]) and *φ* denotes the weight fraction of PVDF in a mixture. The relative crystallinity was estimated to be 92.64% for SPE 80 °C 90 s, 73.24% for SPE 120 °C 90 s, 57.51% for SPE 120 °C 210 s and 44.55% for SPE 160 °C 600 s. The PVDF/NMP/[EMIM][TFSI] electrolytes under the test exhibited lower $\gamma_{c}$ as intensity of solvent evaporation increases during the preparation process. Thus, the SPE prepared at conditions 160 °C for time 600 s is the most amorphous.

### 3.4. X-ray Photoelectron Spectroscopy

**Figure 6:**
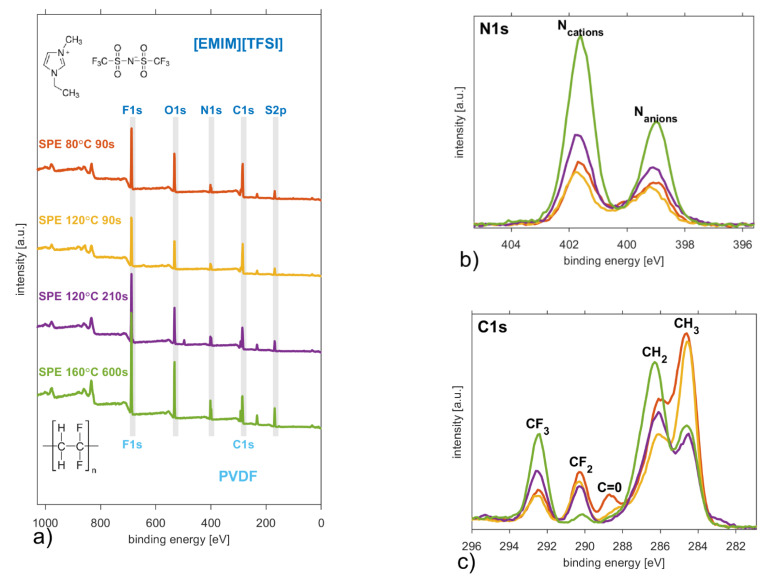
(**a**) survey XP spectrum, (**b**) N1s XP spectrum and (**c**) C1s XP spectrum of the solid polymer electrolytes prepared in four treatment conditions.

All types of the solid polymer electrolytes were subjected to XPS measurement, which provides an estimate of the surface elemental compositions and chemical structures in the samples. An incorrect referencing of the binding energy scale may mislead the interpretation of the data, thus we were cautious about the conclusions drawn from XPS characterization [66]. All XPS binding energies were corrected using the C1s line of (C–C/CH_3_) at 284.6 eV. Figure 6a shows wide range XP spectra for all samples where the expected elements (in PVDF, NMP and ionic liquid) F, N, O, S, and C were detected and confirmed that the mixtures of ionic liquid, polymer matrix and did not produce a new binding energy.

While the F1s and O1s peaks contain a single narrow peak with a full width at half maximum below 1.8 eV, the others (S2p, N1s and C1s) contain distinguishable components. XPS S2p spectra contain only a characteristic doublet peak of the TFSI anion [67,68]. Figure 6b shows how XPS N1s spectra are split two recognisable peaks, where the larger one at 401.7 eV is related to N located at the [EMIM] cation ring and the smaller one at 399.1 eV corresponds to the N anions [69]. Their relative peak surface ratio changes from 1:1.18 (SPE 80 °C 90 s) up to 1:1.75 (SPE 160 °C 600 s), but it never gains the value characteristic for pure ionic liquid [EMIM][TFSI] 1:1.90 published elsewhere [70].

Figure 6c shows that the SPEs have four specific peaks at 292.4 eV (CF_3_), 290.3 eV (CF_2_), 286.1 eV (C–N/CH_2_) and 284.6 eV (C–C/CH_3_). The relatively low boiling point of the solvent NMP [18,34] results in a decrease of NMP content in the SPE mixture and the disappearance of the specific peak at 288.7 eV (C=O) [71,72], which is distinguishable only in the case of the SPE layer prepared at 80 °C for 90 s. Table 2 illustrates the solvent evaporation as the relative chemical compositions change with the thermal and time conditions in the preparation process of different SPEs. The relative amount increases for elements F, N, and S, while it decreases for C as the solvent evaporation rate grows. The relative concentrations of each element were calculated by taking into account the corresponding relative sensitivity factors [73] of our apparatus. Furthermore, the relative intensities of specific peaks become insignificant at 292.4 eV (CF_2_) and decrease at 284.6 eV (C–C/CH_3_) in comparison to the CF_3_ peak. The shape of the C1s XP spectrum becomes more similar to the C1s spectrum of pure ionic liquid [EMIM][TFSI] [70] as more solvent is evaporated. Figure 6c illustrates that the chemical composition of the surface is closer to the composition of the ionic liquid as spherulites grow due to solvent evaporation during the preparation process. This confirms our observations obtained from Raman spectroscopy about ionic liquids being on the surfaces of the spherulites.

### 3.5. Secondary Ion Mass Spectroscopy 

**Figure 7:**
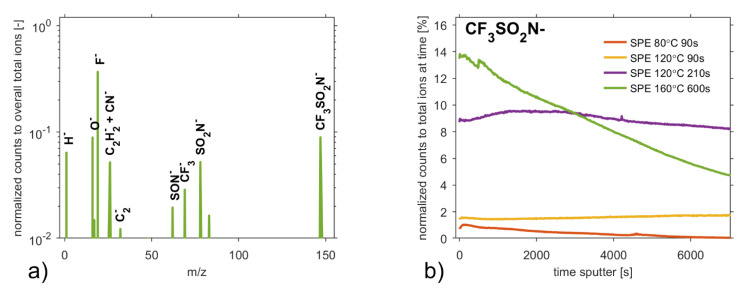
(**a**) Normalized negative ToF-SIMS spectrum taken from a SPE 160 °C 600 s. Counts are normalized to the total sum of counts across the spectrum. Normalized intensities below 0.01 are ignored. (**b**) Normalized depth profiles of CF_3_SO_2_N^−^ anion to total ions at the specific sputter time for all SPEs. Depth profiles are normalized with respect to the total count at specific sputter time. The spectrum and profiles are obtained by employing Cs^+^ ions as a primary projectile with a kinetic energy of 2 keV.

To illustrate the change of the relative concentration of elements over depth, time-of-flight secondary ion mass spectroscopy (ToF-SIMS) was applied to examine the surface of the samples. The negative ToF-SIMS spectra of all the samples exhibit expected peaks which are in agreement with the negative spectra of the ionic liquid [74,75,76] and PVDF [77]. Figure 7a shows that the negative ToF-SIMS spectra are dominated by F^−^ (*m/z* = 19) and O^−^ (*m/z* = 16). Other intensive peaks are characteristic peaks for PVDF (C_2_^−^, CF_2_^−^) or smaller fragments of the [TFSI]^−^ anion, such as CF_3_^−^ at *m/z* = 69, CF_3_SO_2_N^−^ at *m/z* = 147, NSO_2_^−^ at *m/z* = 78 and SO_2_NSO_2_^–^ at *m/z* = 142. The relative intensity of ion fragments of [TFSI]^−^ moiety from a sample surface is assumed to be almost unchanged [76] considering the temperatures of the particular preparation process of the SPEs.

Figure 7b indirectly illustrates the depth profiles of ionic liquid from the surface of particular SPEs on normalized depth profiles of the single-bond fraction (CF_3_SO_2_N^−^) of [TFSI]^−^ anion (*m/z* = 280), which was not detected due to the setting limitations in our measurements. Depth profiles are normalized with respect to total count at a specific sputter time. Figure 7b indicates that the concentration of ionic liquid in SPEs grows with the intensity of the thermal treatment. The depth profiles are the results of integral information over an area of 100 × 100 μm^2^ where the average diameters of the spherical particles are from ~3.08 μm up to ~15.12 μm.

## 4. Conclusions

This work investigates how thermal treatment conditions of PVDF/NMP/[EMIM][TFSI] electrolyte affect its surface morphology, phase composition and chemical composition. The ionic liquid, polymer matrix and solvent were mixed in a weight ratio 1:1:3. After deposition by drop casting on the electrode platform, mixtures were held at (i) 80 °C for 90 s, (ii) 120 °C for 90 s, (iii) 120 °C for 210 s and (iv) 160 °C for 600 s, to get four types of solid polymer electrolyte. These electrolytes consist of spherulites whose average diameter increases with the preparation temperature. Raman mapping shows that the spherulites have a semicrystalline structure and the area between them is amorphous. The crystalline structure is of PVDF α-, β- and γ-phases. Normalized Raman spectra also showed that the peak band of β-phase of PVDF (840 cm^−1^) increases with the intensity of solvent evaporation in the SPE during the preparation process. In other words, the crystallinity is connected to the preparation conditions of the SPE thermal treatment. 

Analysis of FTIR spectra confirmed the results obtained from Raman spectroscopy that β-phase becomes more dominant over other phases. Comparison of these two spectroscopic techniques shows that the positions of particular peaks remained relatively unchanged indicating that polymer matrix (PVDF) and ionic liquid [EMIM][TFSI] have good miscibility in solvent NMP. 

DSC described the decrease of the PVDF crystallinity in PVDF/NMP/[EMIM][TFSI] electrolytes as intensity of solvent evaporation increases during the preparation process.

Relative chemical compositions investigated by XPS increase for elements F, N, and S, while they decrease for C as the solvent evaporation rate grows. For SPE samples with a higher solvent evaporation rate, the shape of the C1s XP spectrum is comparable to the C1s spectrum of the ionic liquid, thus, we could conclude that the chemical composition of the surface is closer to the composition of the ionic liquid as was also observed by Raman mapping at band 741 cm^−1^. 

Taking into account the already published fact that the DC conductivity of the SPE increases with the intensity of the heat treatment and this DC conductivity becomes comparable to the DC conductivity of the ionic liquid, it can be concluded that ion transport takes place predominantly on the surfaces of the spherulites as well as in amorphous regions. 

## Figures and Tables

**Table 1:** Relative percentage of γ- and β-phases in sample considering number of electroactive phases consisting only of γ- and β-phases (100%).

Sample	SPE 80 °C 90 s	SPE 120 °C 90 s	SPE 120 °C 210 s	SPE 160 °C 600 s
β-phase, %	84.11	56.23	73.03	98.80
γ-phase, %	15.89	43.77	26.97	1.20

**Table 2:** Relative chemical compositions determined by XPS.

Sample	Element Content [%]				
	S2p	C1s	N1s	O1s	F1s
SPE 80 °C 90 s	3.28	54.15	5.75	16.02	20.79
SPE 120 °C 90 s	3.44	53.24	5.68	14.91	22.72
SPE 120 °C 210 s	5.97	45.02	9.25	14.96	24.80
SPE 160 °C 600 s	6.94	37.53	11.12	17.91	26.50

## Data Availability

The datasets measured and analyzed during the current study are available from the corresponding author on reasonable request.
